# The Epithelial Sodium Channel (ENaC) Establishes a Trafficking Vesicle Pool Responsible for Its Regulation

**DOI:** 10.1371/journal.pone.0046593

**Published:** 2012-09-28

**Authors:** Robert S. Edinger, Carol A. Bertrand, Christine Rondandino, Gerard A. Apodaca, John P. Johnson, Michael B. Butterworth

**Affiliations:** 1 Department of Medicine, Renal-Electrolyte Division, School of Medicine, University of Pittsburgh, Pittsburgh, Pennsylvania, United States of America; 2 Department of Cell Biology, School of Medicine, University of Pittsburgh, Pittsburgh, Pennsylvania, United States of America; Emory University, United States of America

## Abstract

The epithelial sodium channel (ENaC) is the rate-limiting step for sodium reabsorption across tight epithelia. Cyclic-AMP (cAMP) stimulation promotes ENaC trafficking to the apical surface to increase channel number and transcellular Na^+^ transport. Removal of corticosteroid supplementation in a cultured cortical collecting duct cell line reduced ENaC expression. Concurrently, the number of vesicles trafficked in response to cAMP stimulation, as measured by a change in membrane capacitance, also decreased. Stimulation with aldosterone restored both the basal and cAMP-stimulated ENaC activity and increased the number of exocytosed vesicles. Knocking down ENaC directly decreased both the cAMP-stimulated short-circuit current and capacitance response in the presence of aldosterone. However, constitutive apical recycling of the Immunoglobulin A receptor was unaffected by alterations in ENaC expression or trafficking. Fischer Rat Thyroid cells, transfected with α,β,γ-mENaC had a significantly greater membrane capacitance response to cAMP stimulation compared to non-ENaC controls. Finally, immunofluorescent labeling and quantitation revealed a smaller number of vesicles in cells where ENaC expression was reduced. These findings indicate that ENaC is not a passive passenger in regulated epithelial vesicle trafficking, but plays a role in establishing and maintaining the pool of vesicles that respond to cAMP stimulation.

## Introduction

There is a tightly organized regulation of membrane proteins in polarized cells that helps to establish and maintain polarity and facilitate vectoral responses to internal and external cues. The extensive studies involving both neurons and epithelia demonstrate a degree of similarity in their ability to differentially organize proteins to specific membrane locations [1], [2]. In epithelial cells distinct apical and basolateral membrane domains are maintained by junctional proteins that separate transport and regulatory proteins and organize proteins to these different membrane locations [3]. Like a number of other epithelial ion channels, the epithelial sodium channel (ENaC) is trafficked and faithfully delivered to the apical membrane of epithelial cells in which it is expressed [4]–[7].

The intracellular mechanisms involved in ENaC's regulation by trafficking have been recently reviewed [5], [8], [9]. ENaC is delivered to the apical membrane via the biosynthetic pathway in two forms, both proteolytically cleaved (fully mature/active) and uncleaved (unprocessed) [10]–[15]. Once ENaC is delivered and inserted into the apical membrane a defined path has been described for the channel's internalization and recycling [16]–[21]. In previous work we extensively characterized the trafficking of ENaC in a model mouse cortical collecting duct (mpkCCD_c14_) cell line to demonstrate the role of an intracellular storage pool that was mobilized by cAMP stimulation to increase ENaC density in the apical surface of the cells [22].

ENaC is retrieved from the apical membrane via clathrin mediated endocytosis in a process dependent on ubiquitylation of the channel [23]–[26]. ENaC then traffics to EEA1 (early endosome antigen 1)-positive early endosomes [25]. At this early stage a fate decision is made between degradation and recycling. Some ubiquitylated channels interact with Hrs and ESCRT pathway proteins and are targeted for lysosomal degradation [16] but the majority of ENaC is recycled in the mpkCCD cells, through a Rab11b-positive compartment, to maintain steady-state apical membrane channel number [27], [28]. The role of deubiquitylating enzymes (DUBs) in this recycling has been demonstrated, and we previously investigated the impact of cAMP stimulation on ENaC turnover when DUBs were inhibited [17], [29]. Results from these studies suggested that while ENaC is likely constitutively recycled at the apical membrane, there was a more rapid exocytic delivery and matched endocytic retrieval in the presence of cAMP stimulation.

Here we report that by removing hormonal and steroid supplementation from the cell culture media that the ENaC expression was significantly reduced. In conjunction with the reduction in ENaC expression the trafficking response to cAMP stimulation was also smaller. This cAMP response returned when ENaC expression was restored with replacement of the mineralocorticoid, aldosterone. It was unclear whether the change in vesicle compartment size was due to ENaC expression or some other protein/s that had been induced by aldosterone, so we specifically knocked down ENaC expression while maintaining aldosterone stimulation. Under these conditions the compartment size was again reduced. Inhibiting the activity of ENaC by preventing proteolytic cleavage did not alter the size or responsiveness of the trafficking vesicle pool. Introduction of ENaC into non-native ENaC-expressing epithelia recapitulated this trafficking compartment. These findings in conjunction with the membrane labeling and trafficking assays indicate that ENaC is capable of establishing and maintaining an intracellular vesicle population that is responsive to cAMP stimulation and required to acutely traffic ENaC to the apical surface. This study further differentiates the complex trafficking and recycling regulation observed in polarized epithelial cells from non-polarized and more general cell trafficking models, and underscores the value of performing these studies in polarized models.

## Materials and Methods

### Reagents and Antibodies

All reagents were obtained from Sigma-Aldrich (St. Louis, MO) unless stated. Antibodies used included anti-actin (Sigma), anti-SGK (serum and glucocorticoid-induced kinase) (Cell Signaling Technology, Beverly, MA) and anti-ENaC antibodies (StressMarq, Vicroria, BC, Canada). Membrane labeling was performed using the fixable lipophilic dye, FM1-43FX (Invitrogen). A cell permeant pro-protein convertase inhibitor, furin convertase inhibitor (FCI) (Alexis Biochemical/Enzo, Farmingdale, NY) was reconstituted in sterile water at 100 mM and placed in the culture medium of cells at a final concentration of 100 µM to inhibit the action of intracellular proteases (furin) that proteolytically cleave and activate ENaC.

### Cell Culture

The mpkCCDc14 cells (provided by A. Vandewalle and M. Bens, Institut National de la Santé et de la Recherche Médicale, Paris, France) were grown in flasks (passage 30–40) in defined (supplemented) medium as described previously [22], [28]. Growth medium was composed of equal volumes DMEM and Ham's F12 supplemented with 60 nM sodium selenate, 5 mg/ml transferrin, 2 mM glutamine, 50 nM dexamethasone, 1 nM triiodothyronine, 10 ng/ml epidermal growth factor, 5 g/ml insulin, 20 mM D-glucose, 2% vol/vol FCS, and 20 mM HEPES (Invitrogen, Sigma), pH 7.4, at 37^°^C in 5% CO_2_. The medium was changed every second day. For all experiments, the mpkCCD cells were subcultured onto permeable filter supports (0.4 µm pore size, 0.33 cm^2^ or 4 cm^2^ surface area; Transwell, Corning, Lowell, MA). Cells were cultured in defined (supplemented) medium until a confluent transporting cell monolayer had developed. This was assessed by recording open circuit voltage and transepithelial resistance using “chopstick” electrodes (Millipore, Billerica, MA). Typically, 24 hours before use in any investigation, medium incubating filter-grown cells was replaced with a minimal medium (without drugs or hormones) that contained DMEM and Ham's F12 only. In some experiments, the culture medium was substituted for this minimal (unsupplemented) medium to reduce the expression on ENaC in the cells. Cultures were subsequently stimulated with aldosterone for defined periods (below).

Fisher rat thyroid (FRT) cells (passage 40–60) were maintained in DMEM/F12 media supplemented with 5% FBS, 10 mU/ml TSH, 0.01 mg/ml insulin, 10 nM hydrocortisone, 0.005 mg/ml transferrin, 10 ng/ml somatostatin, 10 ng/ml glycyl-L-histidyl-L-lysine acetate. FRT cultures expressing mouse-ENaC were established by transiently transfecting with 1 µg each of mouse-α,β, and γ ENaC using Lipofectamine 2000 per the manufactures instructions (mouse-ENaC constructs kindly provided by Dr.T.Kleyman, University of Pittsburgh) as previously described [28]. Following transfection, cells were seeded onto 0.33 cm^2^ filter supports (Transwell) and allowed to polarize. Cells were cultured in the presence of 50 nM dexamethasone in the media to maximize ENaC expression and Na^+^ transport. Filter-grown FRT cells were used for short circuit current (I_SC_) measurements 3 days later. Control FRT cells were transfected with equivalent (3 µg total) cDNA encoding the reporter construct eYFP-membrane (Clontech/Invitrogen, Carlsbad, CA) which targets the fluorescent protein to the plasma membrane [28].

### Short-Circuit Current and Membrane Capacitance Recordings

Cells cultured on filter supports were mounted in modified Ussing chambers (Harvard Apparatus, Holliston, MA), and the cultures were continuously short circuited with an automatic voltage clamp in a system that permitted simultaneous recording of short-circuit current (I_SC_) and total membrane capacitance (C_T_) (designed and manufactured by W. Van Driesche KU Leuven, Belgium), using our previously described methods [22](1–3). The bathing Ringer's solution was composed of 120 mM NaCl, 25 mM NaHCO_3_, 3.3 mM KH_2_PO_4_, 0.8 mM K_2_HPO_4_, 1.2 mM MgCl_2_, 1.2 mM CaCl_2_, and 10 mM glucose. Chambers were constantly gassed with a mixture of 95% O_2_/5% CO_2_ at 37^°^C, which maintained the pH at 7.4. A typical cAMP stimulation involved the addition of 10 µM forskolin (Fisher Scientific, Pittsburgh, PA) basolaterally, which produced a maximum I_SC_ stimulation after 10–20 min. To determine the net Na^+^ transport through ENaC, 10 µM amiloride (Sigma) was added to the apical cell surface at the end of each experiment. For experiments using low sodium solutions, the 120 mM NaCl was replaced with N-methyl-D-glucamine chloride (NMDG-Cl, Sigma), as previously reported [22].

### IgA internalization and recycling

The recycling of radiolabelled IgA has been performed previously by us on polarized epithelial cells and described in several studies [30]–[36]. In brief, ^125^I-IgA was iodinated using the ICl method to a specific activity of 1.0–2.0×10^7^ cpm/µg. Filter-grown mpkCCD cells expressing wild-type pIgR were maintained in unsupplemented or fully supplemented media (described above), and ^125^I-IgA was internalized from the apical cell surface of the cells for 10 min at 37°C. Following ligand internalization the apical surface of the cells were treated three times for 10 min with 25 µg/ml trypsin at 4°C to remove cell surface bound ligand. The cells were then treated with 125 µg/ml soybean trypsin inhibitor for 10 min at 4°C. The cells were rapidly washed three times, the apical and basolateral medium aspirated, and replaced with fresh medium. The cells were then incubated for 3 min at 37°C. This wash procedure takes 5 min at 37°C. Fresh medium was added to the cells, and they were chased for up to 2 h at 37°C. At designated time points, the apical and basolateral media (0.5 ml) were collected and replaced with fresh media. After the final time point, filters were cut out of the insert and the amount of ^125^I-IgA quantified with a γ counter. The media samples were precipitated with 10% trichloroacetic acid for 30 min on ice, and then centrifuged in a microcentrifuge for 15 min at 4°C. The amount of ^125^I-IgA in the trichloroacetic acid-soluble (degraded) and insoluble fractions (intact) was quantified with a γ counter. An equal number of cells (not expressing the pIgR) were treated identically, and these values were subtracted from those cells expressing the pIgR. The extent of apical recycling was expressed as a percentage of apical signal at time 0 for cells cultured in supplemented versus unsupplemented medium.

### Apical membrane labeling and vesicle endocytosis

To specifically label apical membrane mpkCCD cells cultured on filter supports were first washed in PBS containing 0.1 mM CaCl_2_ and 1 mM MgCl_2_ (PBS+CM) and then in a Ringer's solution (as above). The apical solution was replaced with Ringers solution containing FM1-43-FX at a concentration of 50 µg/ml which remained on the cells for 0, 2, 5 or 10 minutes. Cells were either maintained under basal (unstimulated) conditions or were stimulated with forskolin (10 µM). At the chosen time points, cells were rapidly washed four times in cold (4^°^C) Ringer's solution to remove excess dye and wash off remaining apical membrane label (that had not been internalized) and immediately fixed in a cold (4^°^C) paraformaldehyde buffer (4% in PBS at pH7.4). Following 30 min fixation at 4^°^C, cells were washed in cold PBS+CM three times, and nuclei were counterstained using Hoechst 33342 (trihydrochloride, 10 nM) cell permeant nuclear dye (Invitrogen) for 10 min. Cells were washed thrice in PBS+CM and mounted onto slides using Fluoromount-G (SouthernBiotech, Birmingham, AL) for imaging, as previously described [28]. Images were captured using an Olympus IX81 fluorescent microscope (Olympus, Center Valley, PA) fitted with a DSU spinning disk and 300 W fluorescent light source using a 60X, 1.4 N.A. oil objective. Single fluorescent images were captured using a Retiga cooled CCD camera (QImaging, Surrey, BC, Canada) at 1024X1024 resolution using SlideBook (Olympus). Linear adjustments of brightness and contrast were made offline in MetaMorph (Molecular Devices Corp, Downingtown, PA). 3-D reconstructions of spinning disk optical sections were carried out using MetaMorph. Vesicle counts from the image stacks were obtained after image threshold and object counting in MetaMorph, and an average vesicle count through the whole image stack was calculated for >50 cells for at least 2 fields on each slide. The vesicle counts for 3 separate experiments were averaged to produce a vesicle number per cell (based on nuclei labeling).

### siRNA

To knock down the expression of βENaC, siRNA was commercially obtained (Dharmacon/Thermo Fisher Scientific, On-Target Plus) and a pool of 4-siRNAs were introduced into the mpkCCD cells using lipofectamine 2000 at a concentration of 50 nM as described previously [17], [28](3). The target sequences for βENaC were GCUCCGAUGUUGCCAUAAA, GCCAUGUGGUUCCUGCUUA, CAUCGGAACUUCACGCCUA and GACCAGAGCUCGAAUAUCA. Cells were seeded onto filter supports and allowed to polarize over 72 hrs before use in electrophysiological experiments. Following I_SC_ and C_T_ measurements the cells were harvested from the filter supports in lysis buffer (62.5 mM EDTA, 150 mM NaCL, 50 mM Tris-HCl, 0.7% Triton-X100, 1.5%NP40, pH 8, containing protease inhibitors) and proteins were resolved on 6–18% SDS-PAGE gels, transferred to PVDF (1 hr at 100 V), and blotted for proteins of interest to determine the extent of protein knockdown. Desitometric quantification of protein band intensities was carried out in Adobe Photoshop CS5.1 (Adobe, San Jose, CA) and values were expressed as a percentage of control signal, following background subtraction, and normalization to total protein expression (actin).

### Statistics

All data were analyzed using SigmaPlot (Systat, Chicago, IL). Summarized data were evaluated for normality and equal variance, and *t*-tests were carried out to determine whether differences were statistically different from each other. For any difference in the mean values, *P*<0.05 was considered significantly different.

## Results

### ENaC Expression and I_Na_ decreases without mineralocorticoid supplementation

**Figure 1:**
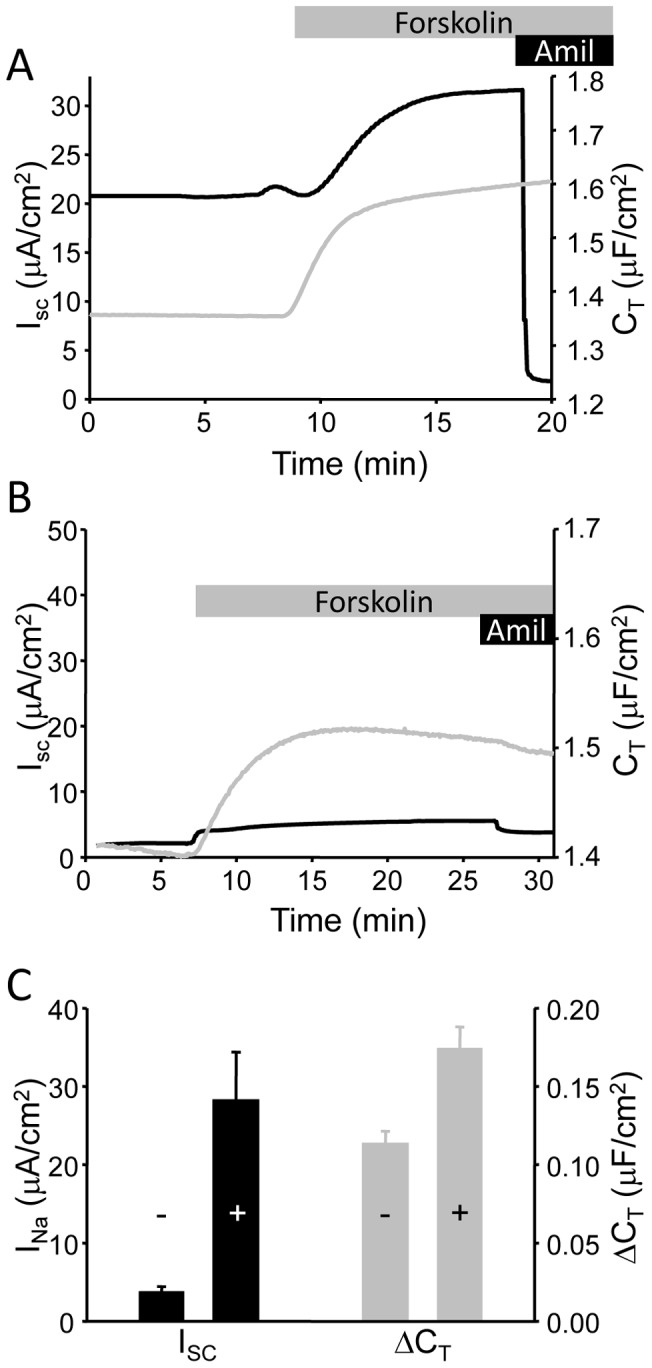
I_SC_ and C_T_ recordings with and without hormonal supplementation. (A) Representative I_SC_ (black trace) and C_T_ (grey trace) recording from mpkCCD cells mounted in modified Ussing chambers and stimulated with (10 µM) forskolin. Addition of 10 µM amiloride at the end of the trace demonstrated the majority of the recorded I_SC_ was Na^+^ transport via ENaC. (B) A similar trace from mpkCCD cells cultured in the absence of dexamethasone supplementation. (C) Summarized data for stimulated amiloride-sensitive current (I_Na_) and C_T_ response to forskolin stimulation (n = 14) in cells with (+) and without (-) full supplementation.

Figure 1A illustrates typical short-circuit current (I_SC_) and transepithelial capacitance (C_T_) traces from mpkCCD epithelia cultured in fully supplemented media and stimulated with 10 µM forskolin. The complete media used to culture the mkpCCD cells is supplemented with a mixture of hormones and trace metals which includes 50 nM dexamethasone (dex) to maximize ENaC expression (see methods). The addition of forskolin (10 µM) to the basolateral bathing chamber induces an increase in measured I_SC_ and C_T_. We previously characterized this response to show the I_SC_ increase was due to the insertion of ENaC-containing vesicles into the apical membrane, which increased both channel number (as determined by surface biotinylation) and apical membrane surface area as measured by membrane capacitance (C_T_) [22], [37]. When the cells are cultured in an unsupplemented medium (as described in the methods) without hormones or steroids, ENaC expression is reduced (Figure 1B) and the amiloride-sensitive I_SC_ is significantly smaller under both basal and cAMP-stimulated conditions (Figure 1B). In conjunction with this loss in ENaC-mediated current there is a reduction in the membrane capacitance response with forskolin stimulation (Figure 1B, and summarized in Figure 1C). The reduction in ΔC_T_ stimulation in the absence of dex supplementation suggests a smaller number of vesicles were exocytosed in response to cAMP stimulation along with the reduction in ENaC expression.

### Aldosterone restores ENaC expression and cAMP response

**Figure 2:**
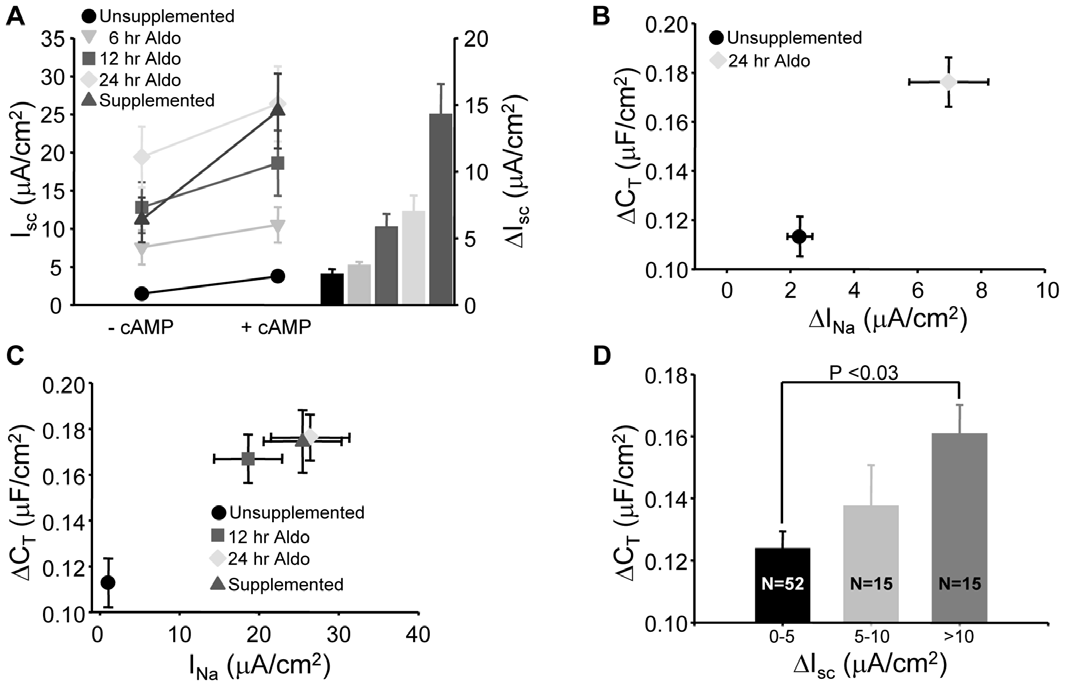
Aldosterone restores I_SC_ and C_T_ responses to cAMP stimulation. (A) Cells in unsupplemented media were incubated with 100 nM aldosterone for increasing time and stimulated with forskolin. The basal (-cAMP) and forskolin stimulated (+cAMP) currents from cells with no aldosterone (unstimulated), 6, 12 and 24 hr aldosterone stimulation were compared to cells which received fully supplemented medium (supplemented). The average I_SC_ response to cAMP stimulation is plotted as a bar graph on the right (n = 16). (B) The change in capacitance (ΔC_T_) versus change in amiloride-sensitive I_SC_ (ΔI_Na_) in response to forskolin stimulation is summarized for cells without supplementation and 24 hr stimulation with aldosterone. Both I_SC_ and C_T_ responses were significantly greater in the aldosterone treated cells compared to unsupplemented controls (n = 16, p<0.01). (C) The change in capacitance versus total stimulated ENaC I_SC_. The I_Na_ and ΔC_T_ of the fully supplemented cells was not significantly different from cells treated with aldosterone for either 12 hr or 24 hrs, but these 3 groups were significantly greater than cells that received no supplementation (p<0.01). (D) Data for all recordings were pooled (regardless of supplemented state) and categorized into groups that responded to cAMP with 0–5, 5–10 and >10 µA/cm^2^ΔI_SC_. The average ΔC_T_ was then plotted for each group. A significantly larger capacitance response was observed in cells with ΔI_SC_ greater than 10 µA/cm^2^ compared to those with less than 5 µA/cm^2^.

To examine the possibility that the cells cultured in basic media were primarily affected by the removal of steroids from the media, they were treated with increasing concentrations and over increasing time with aldosterone (Figure 2A). A significant increase in ENaC-mediated I_SC_ was observed after 6 hours of aldosterone treatment and by 12 hours both basal and cAMP stimulated I_SC_ had returned to control levels (Figure 2A,C). The maximal increase in I_SC_ was typically observed by 24 hours with no significant increase in ENaC currents after 24 hours or above concentrations of 100 nM. We previously reported on the time course of aldosterone (aldo) stimulation in the mpkCCD cells and these I_SC_ results are in agreement with the previously published results [38], [39]. All subsequent stimulation experiments therefore employed a 100 nM, 24 hour aldo stimulation.

In conjunction with the increase in amiloride-sensitive I_SC_ with aldo stimulation, the change in C_T_ with forskolin stimulation also increased. After a 24 hour aldo stimulation the ΔC_T_ and ΔI_SC_ response to cAMP stimulation were significantly greater than cells that remained in unsupplemented medium (Figure 2B). Therefore as ENaC expression increased there was a larger intracellular vesicle pool of ENaC available for insertion in to the apical membrane after cAMP stimulation (Figure 2C). By combining the data for all recordings, in both supplemented and unsupplemented media, the summarized graph (Figure 2D) indicates a smaller ΔI_SC_ response to forskolin is associated with a reduced capacitance response.

### ENaC knockdown reduces the cAMP-induced capacitance increase

**Figure 3:**
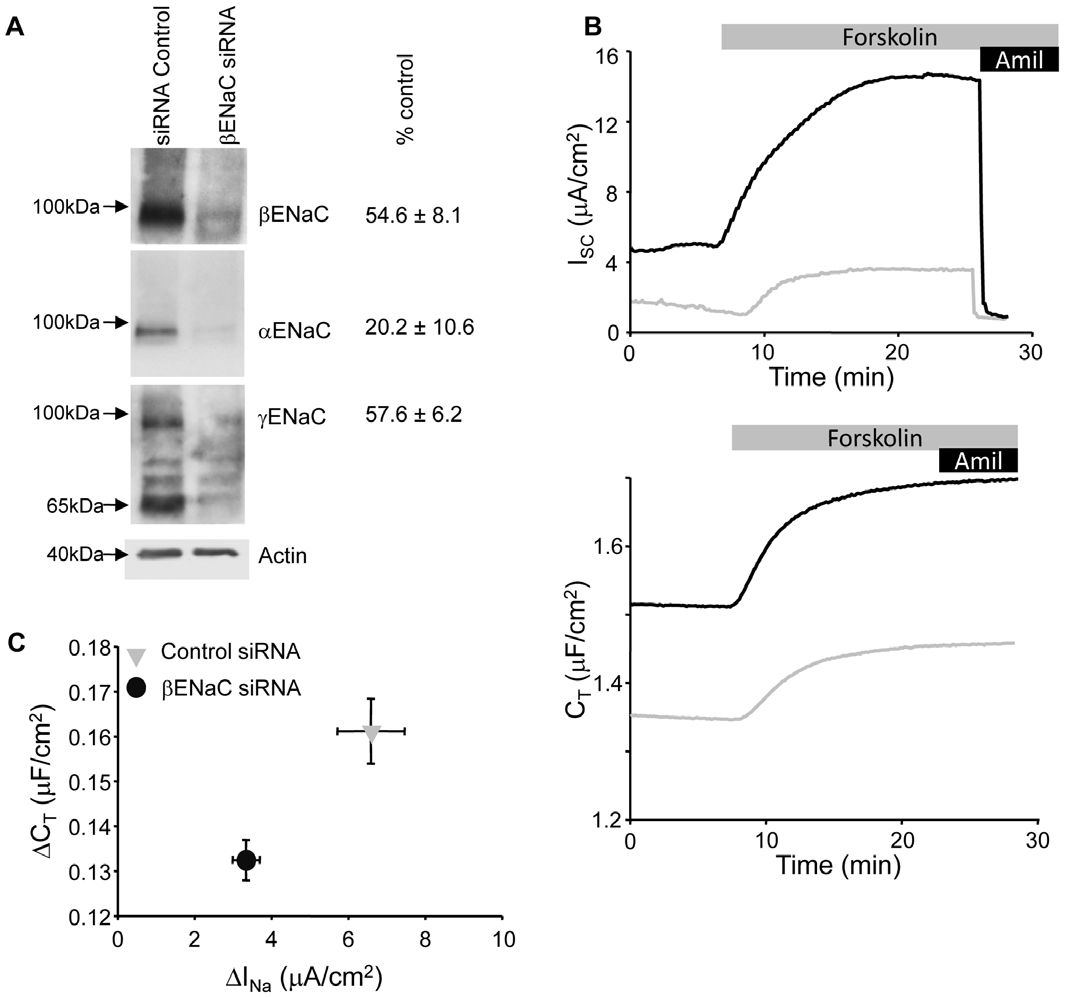
ENaC siRNA reduces I_SC_ and cAMP C_T_ response. (A) Western blots of whole cell lysate from βENaC knockdown demonstrate reduction in expression of ENaC compared to control siRNA transfected mpkCCD cells. The actin-corrected percent reduction in expression (n = 3) are summarized to the right of each representative blot. (B) Representative traces for I_SC_ (top traces) and C_T_ recordings (bottom) for control (black traces) and ENaC knockdown (grey traces) cells stimulated with forskolin (10 µM). (C) Summarized data for I_SC_ versus C_T_ responses to forskolin stimulation for control (n = 45) and βENaC (n = 55) knockdown cells similar to those presented in (B).

As the expression of ENaC was altered by changing the steroid hormone supplementation of the cells it was possible that the change in C_T_ response was due to the alteration in expression of an aldosterone-induced protein involved in vesicle trafficking and not ENaC itself. To directly test if ENaC mediates the C_T_ response we used an RNAi knockdown approach to reduce the expression of βENaC in the mpkCCD cells cultured in the presence of aldo. The knockdown of βENaC resulted in a reduction of all three ENaC subunits (Figure 3A), and a decline in both basal and cAMP-stimulated amiloride-sensitive I_SC_ (sample traces Figure 3B). The loss of ENaC-mediated Na^+^ transport was paralleled by a reduction in the basal and cAMP-stimulated C_T_ (Figure 3B). A summary of a number of similar experiments is presented in Figure 3C which plots the I_SC_ and C_T_ responses to cAMP stimulation.

### Reducing ENaC activity does not disrupt vesicle trafficking

**Figure 4:**
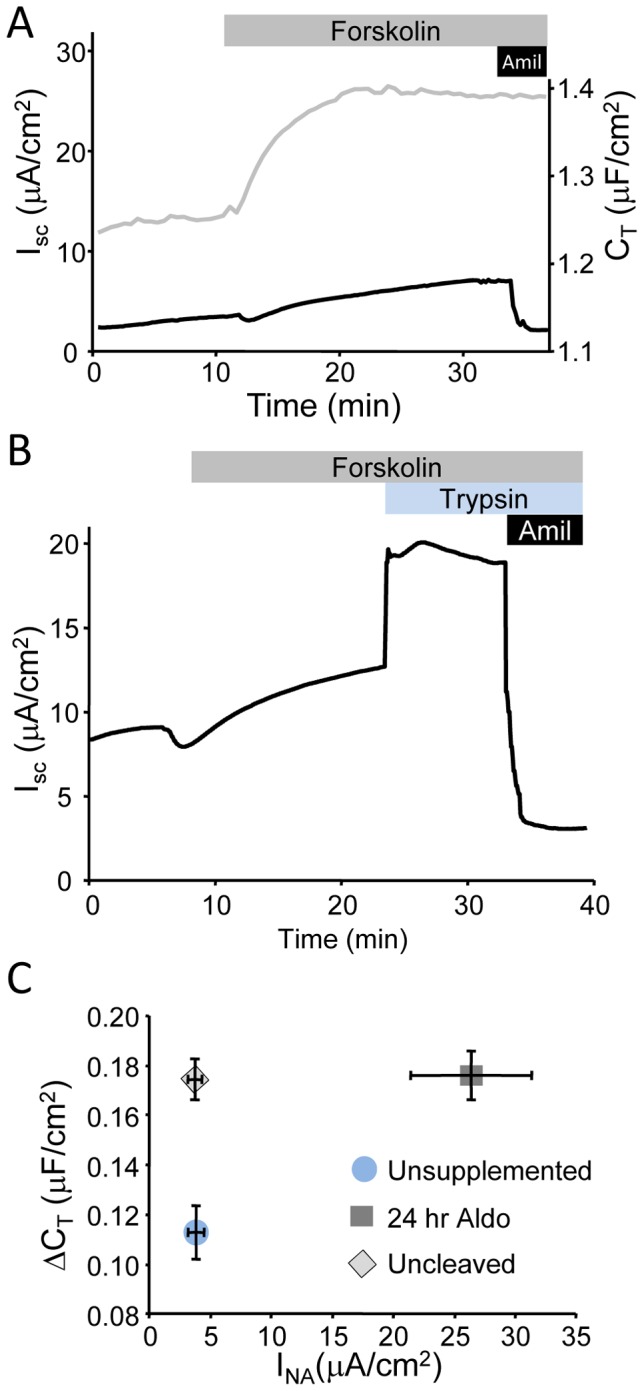
Inhibiting ENaC activity does not alter trafficking. (A) Representative I_SC_ (black trace) and C_T_ (grey trace) traces from mpkCCD cells pretreated with 50 µM FCI for 24 hrs before recordings and stimulated with forskolin (10 µM). (B) Single I_SC_ recording from FCI inhibited cells (as in A) where 1 µM trypsin was added to the apical bath (light blue bar) to activate uncleaved channels. (C) Summarized I_SC_ versus ΔC_T_ plots for control unsupplemented, aldosterone treated and aldo+FCI treated cells (n>16 per point). The ΔC_T_ for uncleaved channels was significantly greater (p<0.01) than the unsupplemented controls, but not significantly different from aldosterone stimulated cells.

While the knockdown results suggested that the number of ENaCs expressed were responsible for establishing the size of the vesicle pool trafficked in response to cAMP signaling, we next sought to determine if the activity or cleavage state of ENaC altered the capacitance response to cAMP stimulation. A number of proteases have been reported to cleave both α- and γ-ENaC and are required to achieve a fully active channel and maximal sodium transport [10], [11], [24], [40]–[53]. Recent reports have suggested that cleaved ENaC may be regulated differently from the uncleaved channels [54]. We blocked full proteolytic cleavage of ENaC by using a pro-protein convertase inhibitor FCI. In cells where the activity of ENaC had been reduced by preventing full proteolytic activation, both basal and stimulated I_SC_ was reduced (Figure 4A). The reduction in I_SC_ was similar to that observed in βENaC knockdown or cells cultured in unsupplemented medium (compare Figure 4C with Figure 3C). However, unlike with βENaC knockdown, the change in C_T_ with cAMP stimulation was not significantly altered by the reduction in ENaC activity. To confirm the presence of uncleaved ENaC at the apical surface of mpkCCD cells, trypsin (1 µM) was added to the apical Ussing hemi-chamber to acutely activate uncleaved ENaC following cAMP stimulation, (see sample trace Figure 4B). A rapid increase in I_SC_ was observed following trypsin addition indicating that a pool of uncleaved ENaC was present in the apical membrane. The summary plot of changes in I_Na_ against the change in C_T_ is presented in Figure 4C.

These data indicate that it is the expression rather than the activity or cleavage state of ENaC that determines the size of the vesicle pool which responds to cAMP stimulation. Sodium transport through ENaC does not impact the C_T_ recording. As confirmation of this, the addition of amiloride at the end of each trace to block ENaC does not significantly alter the C_T_ recordings (see all sample traces), indicating that the C_T_ changes recorded are the result of changes to the membrane surface area which is not influenced by ENaC activity.

### ENaC expression in FRT cells induces a trafficking vesicle population

As knockdown of ENaC reduced the C_T_ response to cAMP stimulation suggesting that ENaC expression was regulating the size of this vesicle pool, it was reasonable to hypothesize that introduction of ENaC into epithelial cells which do not highly express the channel may induce the formation of a trafficking vesicle pool. To test this we employed the Fisher Rat Thyroid (FRT) epithelial cell line which has been used previously by a number of researchers to investigate the function and trafficking of ENaC [14], [54]–[57]. These cells do not natively express detectible levels of α,β,γ-ENaC, are readily transfected to express functional ENaC. The FRT cells can be cultured on filter supports where they form a polarized transporting epithelial monolayer.

**Figure 5:**
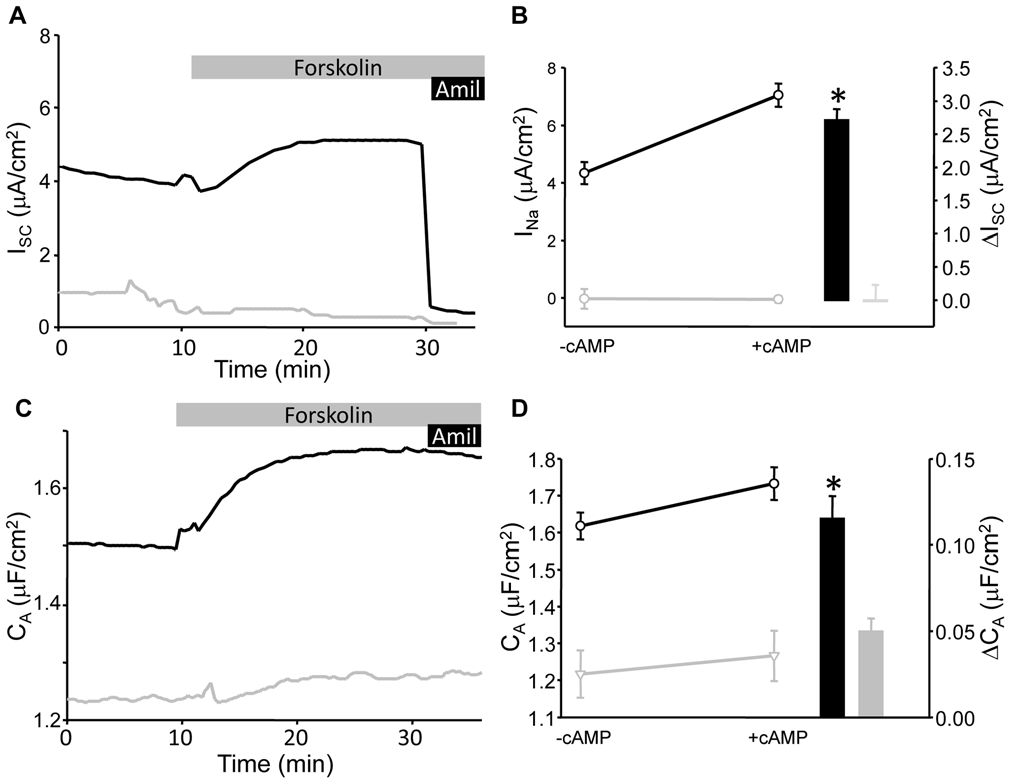
ENaC expression in FRT cells . (A) Representative I_SC_ trace from FTR epithelial cells transfected with α,β,γENaC (black trace) or control plasmid (grey trace), mounted in Ussing chambers and stimulated with 10 µM forskolin (grey bar). The expression of ENaC was confirmed by the addition of 10 µM amiloride (black bar) at the end of the recording. (B) Data from a number of similar experiments (n = 34) are summarized. Basal (-cAMP) and forskolin-stimulated (+cAMP) I_Na_ are presented on the left and the change in I_SC_ with foskolin stimulation (ΔI_SC_) on the right (bar graph). (C) Representative capacitance trace from the same samples provided in (A). (D) Summarized data for capacitance changes under basal and forskolin-stimulated conditions as in (B). (* - indicates significantly different from untransfected p<0.05).

To investigate the capacitance changes occurring at the apical surface of control and ENaC transfected FRT cells, filters were mounted in the Ussing chambers in an apical to basolateral Na^+^ gradient and the basolateral membranes permeabilized using 50 µM nystatin. We have employed this technique previously to isolate membrane domains and record changes in capacitance at the apical membrane alone [22]. Under these conditions a readily detectible amiloride-sensitive I_SC_ was observed in ENaC transfected cells which could be stimulated by forskolin and fully inhibited by the specific ENaC blocker amiloride (Figure 5A). As the basolateral membranes were permeabilized, capacitance measurements reflected only changes occurring in the apical membrane (C_A_). ENaC-dependent current was absent in the control cells (see traces Figure 5A and summary Figure 5C). A small apical capacitance increase was recorded in control cells following cAMP stimulation (Figure 5C,D). The C_A_ response was however significantly greater in FRT cells transfected with α,β,γ-ENaC compared to non-ENaC expressing controls indicating a larger pool of vesicles was trafficked in response to cAMP stimulation.

### Constitutive recycling is not impacted by altering ENaC expression

We have previously demonstrated that the increase in ENaC activity and C_T_ response to cAMP stimulation in mpkCCD cells is representative of a regulated recycling of the channel [22]. Withdrawal of forskolin results in a reversal of I_SC_ and C_T_ responses and cells can be repeatedly challenged with cAMP to elicit rounds of insertion and retrieval of ENaC [22]. Studies with cycloheximide inhibition of new channel synthesis and specific apical blockers have demonstrated that repetitive stimulations involve recycling of the apical channels rather than supplementation from the biosynthetic pool [22]. Here, we noted that while ENaC expression altered the size of the vesicle pool exocytosed in response to cAMP stimulation, the capacitance response is not entirely eliminated when ENaC expression was reduced by a removal of hormonal supplementation or specific siRNA knockdown. The cAMP stimulation still induced an increase in C_T_ in unsupplemented mpkCCD or ENaC knockdown cells. There was also a small C_T_ response in the untransfected FRT cells. It is therefore likely that an apical recycling vesicle compartment remains intact in the absence of ENaC expression. To test this we investigated the recycling of IgA in the mpkCCD cells.

Transferrin receptor or IgA recycling is often employed to investigate the mechanics of cargo turnover in model cell lines. As transferrin receptors are not expressed on the apical surface of mpkCCD cells, we chose to investigate the constitutive recycling of IgA by using the polymeric immunoglobulin receptor (pIgR). pIgR was transfected into mpkCCD cells and radiolabeled IgA used to assay the apical recycling of pIgR in cells with and without aldosterone supplementation. From the supplemental figure (figure S1) it is clear that the majority of the IgA was recycled back to the apical surface. There was no difference in the rate or extent of IgA recycling in cells without supplementation compared to the fully supplemented cells. These data indicate that constitutive apical recycling remains intact in cells receiving no supplementation, where ENaC expression has been reduced, and that the constitutive apical recycling compartments are unaffected by the alteration in ENaC expression. The cAMP-responsive pool of ENaC appears to reside in a vesicle compartment that is regulated separately from the constitutive apical recycling compartments responsible for IgA recycling.

### Membrane labeling demonstrates a larger vesicle population with ENaC expression

**Figure 6:**
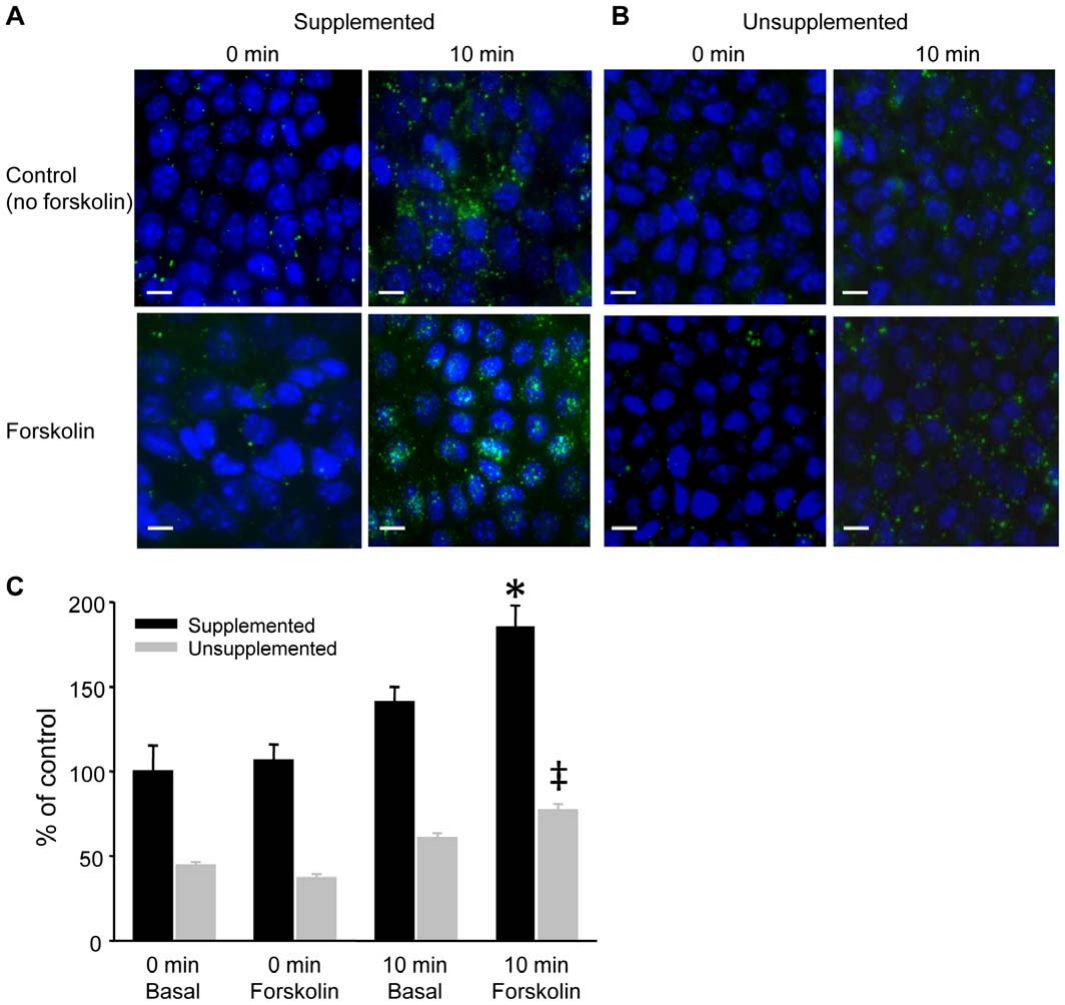
Confocal fluorescent images of FM1-43X endocytosis in mpkCCD cells. (A) Representative maximum projection images from confocal stacks obtained at 0 min and 10 min in basal (no forskolin) and forskolin stimulated (10 µM) cells previously cultured in fully supplemented media. (B) Representative images as in (A) from cells cultured without dexamethasone supplementation. (Bars represent 10 µm). (C) The number of vesicles internalized per cell is presented as a percentage of the fully supplemented counts at time 0 min (N = 3, n>200). Counts from cells cultured without dexamethasone supplementation (Unsupplemented) were significantly lower than cells in full supplementation for all conditions (p<0.05). For fully supplemented cells, there was a significantly greater number of vesicles internalized in the presence of forskolin than from cells without forskolin stimulation after 10 minutes (*, p<0.05). The percent of internalized vesicles in unsupplemented cells stimulated with forskolin was smaller than the equivalent vesicle number at 10 min in cells with full supplementation (‡, p<0.05).

The C_T_ measurements from cells cultured without supplementation indicated a significant decrease in the number of vesicles that traffic ENaC to the apical membrane in response to cAMP stimulation. In order to maintain a steady state membrane surface area, the rate of endocytosis needs to match the exocytic rate. The capacitance recordings indicate that under basal conditions the membrane surface area remains stable. Following the addition of forskolin and cAMP stimulation there is an increase in C_T_ which reaches a plateau by 10 minutes of forskolin stimulation (see sample traces Figure 1A and Figure 3). At this point the C_T_ is again at steady state and thus the numbers of vesicles being internalized must match those being exocytosed. Under steady state conditions it should therefore be possible to use the endocytic rate or number of endocytosed vesicles as a measure of the corresponding vesicle exocytosis. We made use of the fixable membrane labeling fluorophore, FM1-43-FX to label endocytic vesicles. FM1-43-FX was included in the apical bathing medium over increasing time periods under basal conditions (unstimulated), and in the presence of forskolin. Representative confocal images from cells cultured with and without supplementation under these experimental conditions are presented in Figure 6. It is apparent from the images (Figure 6A&B) that there is a reduction in vesicle number in cells cultured in basic media compared to aldosterone stimulated cells. To quantitate the vesicle numbers at each time point, optical sectioning was performed using a spinning disk confocal microscope to produce 3-D images of the cell monolayer. The total vesicle counts were obtained from at least 50 cells in 2 fields of view from 3 separate experiments (average number of cells per time point  =  247.7±14.6) and normalized to control cells without forskolin stimulation at time 0 min (Figure 6C). Two time points are presented, 0 and 10 minutes. There was a significantly smaller number of internalized vesicles in cells cultured without supplementation compared to aldo supplemented cells at all time points and experimental conditions. For aldo supplemented cells, an increase in vesicle number was observed at 10 minutes compared to the 0 minute time point, and a significant increase in vesicle number was observed in cells stimulated by forskolin compared to unstimulated cells. This finding indicates a more rapid vesicle turnover in the presence of forskolin. In all cases, the number of vesicles per cell in unsupplemented cells was significantly reduced compared to cells with supplementation. The data are in agreement with the electrophysiological recordings and support the idea that loss of ENaC expression results in a smaller vesicle pool that traffics to, and from, the apical surface.

## Discussion

ENaC is the rate limiting step in Na^+^ absorption across epithelial tissues in which it is expressed. By altering the activity and density of ENaC in the apical membrane a precise regulation of Na^+^ absorption is achieved. Our previous work detailed the regulated recycling of ENaC at the apical membrane of kidney CCD cells (see ***Introduction***). From the data presented above and our previous observations two distinct populations of trafficking and recycling ENaC can be observed in the mpkCCD cells. First, ENaC appears to be continuously recycled to and from the apical surface under non-stimulated steady-state conditions. Under basal/steady-state conditions, membrane capacitance is unchanged, however if the DUB responsible for removing ubiquitin from endocytosed ENaC is inhibited, an immediate rundown in ENaC-mediated I_SC_ was observed [17]. This observation suggested that while vesicles were still being delivered to the apical surface, ENaC that remained ubiquitylated was moved down a degradative path rather than being recycled. From data obtained in this study, the constitutive turnover of IgA was not impacted by alterations in ENaC expression; however a reduction in basal C_T_ was observed when ENaC expression was reduced. It is possible that ENaC may be recycled constitutively along with other apical surface proteins under unstimulated conditions, but the reduction in basal C_T_ would argue against this.

The population of ENaC that is rapidly recruited to the apical surface by exocytosis appears to be in a compartment that is dynamically regulated by cAMP agonists such as vasopressin or forskolin. An increase in ENaC abundance increases the number of vesicles trafficked and fused with the apical surface as determined using membrane capacitance. Stimulation of cAMP results in two responses. First there is a directed delivery of ENaC from a subapical source. Several lines of evidence point to a reserve population of ENaC that can traffic to the apical membrane in response to cAMP. The apical membrane capacitance increases in parallel with an increase in I_Na_ indicating the exocytosis of ENaC-containing vesicles. Previous studies using biotin labeling demonstrated the delivery of additional channels to the surface following cAMP stimulation [37]. Coupled with this exocytic insertion event is acceleration in the turnover of apical membrane; that is, the rate of endocytosis is accelerated to match the increased rate of exocytosis. Evidence for this can be obtained from our previous study with the DUB inhibitor where the rate of I_SC_ rundown was accelerated in the presence of cAMP [17] and in this study where an increase in the number of endocytosed vesicles in the presence of cAMP stimulation is reported. The capacitance eventually reaches a steady state plateau after about 10 minutes of forskolin stimulation suggesting that the rate of endocytosis matches the accelerated exocytic rate. It is this rapidly mobilized vesicle pool that is regulated by ENaC abundance.

To understand the mechanisms that regulate this recycling compartment we considered at least two possibilities, namely that ENaC would be located in pre-existing recycling compartments and co-regulated with other apical transporters and proteins that are acutely trafficked in response to cAMP. Other apical channels and transporters are being trafficked in kidney epithelia and regulated concurrently with ENaC. In the principal cells of the distal kidney nephron, these include the water channel aquaporin 2 (Aqp2), the urea transporter (UT-A1) and possibly the potassium channel KCNJ1 or ROMK [58]–[70]. These transporters respond acutely to hormonal stimulation or intracellular signaling cascades in a manner similar to that described for ENaC. The action of vasopressin, acting through cAMP is known to induce the trafficking of Aqp2 and UT-A1 to the apical membrane by vesicle translocation [58]. If this trafficking response of multiple transporters to the same agonist involves the movement of vesicles from an intracellular store, as the time-courses suggest, then a fundamental question becomes how these transporters are differentially regulated.

A potential clue to answer this question can be found in the observations that the trafficking response to cAMP stimulation was never entirely eliminated. The number of trafficked vesicles was merely reduced with the loss in ENaC expression. In all cases where ENaC expression was altered an alternative trafficked vesicle pool remained intact. It is likely that these vesicles represent separate compartments involved in the trafficking of other membrane proteins, but this has yet to be determined.

Here we observed that ENaC trafficking and recycling was impacted by reducing ENaC's expression directly. The siRNA results indicate that the specific reduction of ENaC expression, without altering the aldosterone levels, produced the same reduction in the C_T_ response to cAMP stimulation as that observed in unsupplemented cells. This finding opens the possibility that a form of cargo recognition is occurring so that ENaC was able to recruit the necessary accessory proteins and trafficking partners required for its regulation, regardless of whether the expression levels of these accessory proteins change with aldosterone. There is precedent for this type of selective cargo/vesicle interaction [71]–[75]. A similar interaction between ion channel cargo and relative size of the cAMP induced exocytic events has been described for CFTR [76]. This regulatory complex would then be mediated by ENaC itself to allow for the selective trafficking of this channel independent from other transporters destined for the apical surface.

Recent reports have demonstrated differential regulation of ENaC with different cleavage states [77]. We altered the cleaved state of the channel to determine if this would alter the trafficking pathways or impact the vesicle-mediated cAMP response. In addition to investigating how cleavage may impact ENaC's regulation, we verified that the C_T_ recordings were not being altered by changes in Na^+^ conduction through ENaC. While there was a clear reduction in ENaC conductance as recorded by the significant decrease in I_Na_, the C_T_ response to cAMP stimulation was not significantly altered by the inhibition of ENaC activity. The addition of exogenous trypsin confirmed that ENaC was present in the apical membrane. Vesicle trafficking was not significantly impacted by preventing ENaC's proteolytic activation.

We present evidence that ENaC may be regulated in a unique fashion in epithelial cells. While the timing and trafficking kinetics induced by physiological cAMP agonists are similar for ENaC and other apically trafficked transporters, there appears to be a unique subset of vesicles that are responsible for the regulated trafficking of ENaC alone. This compartment adapts to accommodate ENaC numbers and is regulated separately from constitutive apical protein recycling. The presence of such a subset of vesicles allows for the differential regulation of transporters in response to different physiological cues. For example, in the kidney where co-ordinate trafficking of aquaporin and ENaC would be required to allow for directional Na^+^ and water uptake, both transporters could be moved up to the apical surface in response to vasopressin [78]–[80]. Alternatively, if only one or two transporters are required it would offer cells the ability to discriminate the population of vesicles to be trafficked, and this disconnect in ENaC and AQP2 trafficking has been observed *in vivo* [81]. The mechanisms behind such specialized trafficking events remain to be elucidated, and the regulation involved would therefore become increasingly important as we try and understand the cell biology that underlies the observed physiological response.

## Supporting Information

Figure S1 **IgA recycling in mkpCCD cells.** The percent IgA recycled over time for mpkCCD cells in fully supplemented (Supplemented) versus unsupplemented media is provided. There is no significant difference for percentage IgA recycling at any time point.
(TIF)
