# Supplementary figures and images for: The Epithelial Sodium Channel (ENaC) Establishes a Trafficking Vesicle Pool Responsible for Its Regulation
Source: PLoS One. 2012 Sep 28;7(9):e46593. doi: 10.1371/journal.pone.0046593 (PMC3460899; doi:10.1371/journal.pone.0046593)

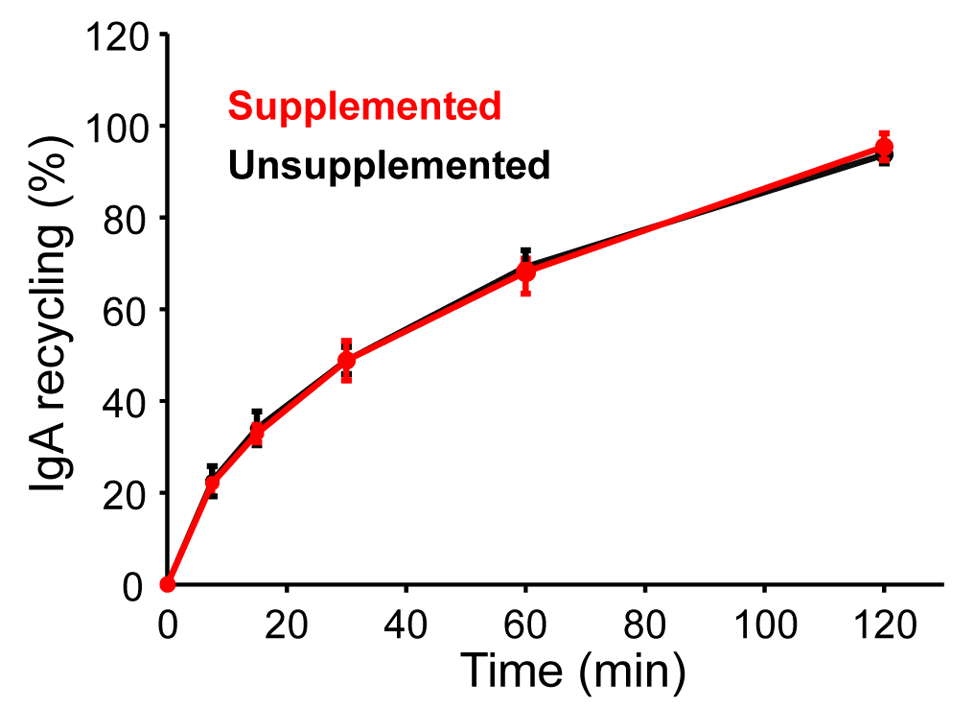

Supplement: Figure S1 — IgA recycling in mkpCCD cells. The percent IgA recycled over time for mpkCCD cells in fully supplemented (Supplemented) versus unsupplemented media is provided. There is no significant difference for percentage IgA recycling at any time point. (TIF) [file pone.0046593.s001.tif]
